# Progress in Surgery

**Published:** 1901-11-30

**Authors:** 


### Progress in Surgery.

### GENITO-URINARY.

**Cystoscopy.**—B. Goldberg (Wildungen)[1] records that he last year performed cystoscopy 150 times, 22 times in females, and 128 times in males. He found disease of the bladder present 85 times and absent 37 times. Of 21 patients with bladder tumours 14 were examined with the cystoscope, several of them more than once. The seven patients not so examined were those in whom the diagnosis was obvious without cystoscopy and an operation was not contemplated. The writer remarks that it is very seldom impossible to use the instrument in cases of tumour. In some cases tumours cannot be diagnosed without cystoscopy, while in others the procedure is necessary for the determination of the extent, position, and form of the tumour. Cystoscopy is generally a necessary preliminary to operation in tumour cases. With regard to stone in the bladder it is pointed out that a lithotrite will determine the friability of a stone which the cystoscope will not do. If the bladder is still free from cystitis sounding for stone is less likely to infect the organ than is cystoscopy. But if the cystoscope is not used in stone cases serious mistakes are likely to be made, especially if tumours and stone exist together, or if incrusted tumours are present. In 10 cases the author found no stone present though stone had been suspected on other grounds. In 55 cases of tuberculous disease of the urinary organs only seven required cystoscopy; of these the bladder was the seat of disease in three cases and the kidney in four. Out of the large number of patients treated for enlarged prostate without complications, the writer only cystoscoped six. It is necessary to use the cystoscope however in these cases if an operation is contemplated. Of the other patients examined fourteen had cystitis, three hypertrophy, one a diverticulum, and one a foreign body. The author catheterised the ureters with the aid of the cystoscope six times, and five times performed intravesical operations. Thirteen times the cystoscope showed that the kidney was diseased and the side affected. He advises the application of a drop of sterilised olive oil and not merely glycerine to the tip and knee of the cystoscope if the urethra is not normal. This renders its introduction easier and does not interfere with vision. If the bladder is intolerant of even gentle syringing, it is recommended to inject 50 ccm. of a 5 per cent. solution of antipyrin into the organ 10 or 20 minutes before washing out and passing the cystoscope. This solution diminishes the bladder irritability more than cocaine, or even than chloroform. If circumstances favour infection, such as the presence of hæmaturia, retention, tumours or tuberculosis, the cystoscopy must be followed by the injection of 100 to 200 ccm. of 1 in 500 to 1 in 1,000 silver nitrate solution. Loewenhardt[2] (Breslau), after describing Pryor's method of cystoscopy, which method was described in a previous issue of The Hospital, makes the interesting remark that those who have explored the ureters with the catheterising cystoscope of Nitze will no longer wish to use the method of direct inspection. Rafin[3] recently stated that the cystoscope was as indispensable to the urological surgeon as the ophthalmoscope to the oculist, and that Albarran, by the adaptation of the cystoscope, had made catheterism of the ureter a simple and reliable operation. He has performed this catheterism seven times. Twice in cases of kidney tumour the sound side was catheterised to estimate the functional condition of the healthy kidney before operation. Once urine was collected from the ureter for bacteriological purposes in suspected tuberculosis. In another case obstruction of the ureter by a calculus was verified. In other cases the object was to discover how displacement of a movable kidney affects the secretion from the organ. The results showed that some degree of renal retention usually resulted from the displacement. Rafin has used both Albarran's and Nitze's cystoscopes. He has not noticed any unpleasant after-effects, and usually finds even local anæsthesia unnecessary.

[1] Centralb. f. Chir., No. 30, July 27, 1901. [2] Centralb. f. Chir., No. 25, June 22, 1901. [3] Vide Abst. Brit. Med. Jour. Epitome, Nov. 2, 1901.

